# Towards achieving Abuja targets: identifying and addressing barriers to access and use of insecticides treated nets among the poorest populations in Kenya

**DOI:** 10.1186/1471-2458-10-137

**Published:** 2010-03-16

**Authors:** Jane Chuma, Vincent Okungu, Janet Ntwiga, Catherine Molyneux

**Affiliations:** 1 Kenya Medical Research Institute-Wellcome Trust Research Programme, Kilifi, Kenya; 2 Centre for Tropical Medicine, Nuffield Department of Clinical Medicine, University of Oxford, Oxford, UK

## Abstract

**Background:**

Ensuring that the poor and vulnerable population benefit from malaria control interventions remains a challenge for malaria endemic countries. Until recently, ownership and use of insecticides treated nets (ITNs) in most countries was low and inequitable, although coverage has increased in countries where free ITN distribution is integrated into mass vaccination campaigns. In Kenya, free ITNs were distributed to children aged below five years in 2006 through two mass campaigns. High and equitable coverage were reported after the campaigns in some districts, although national level coverage remained low, suggesting that understanding barriers to access remains important. This study was conducted to explore barriers to ownership and use of ITNs among the poorest populations before and after the mass campaigns, to identify strategies for improving coverage, and to make recommendations on how increased coverage levels can be sustained.

**Methods:**

The study was conducted in the poorest areas of four malaria endemic districts in Kenya. Multiple data collection methods were applied including: cross-sectional surveys (n = 708 households), 24 focus group discussions and semi-structured interviews with 70 ITN suppliers.

**Results:**

Affordability was reported as a major barrier to access but non-financial barriers were also shown to be important determinants. On the demand side key barriers to access included: mismatch between the types of ITNs supplied through interventions and community preferences; perceptions and beliefs on illness causes; physical location of suppliers and; distrust in free delivery and in the distribution agencies. Key barriers on the supply side included: distance from manufacturers; limited acceptability of ITNs provided through interventions; crowding out of the commercial sector and the price. Infrastructure, information and communication played a central role in promoting or hindering access.

**Conclusions:**

Significant resources have been directed towards addressing affordability barriers through providing free ITNs to vulnerable groups, but the success of these interventions depends largely on the degree to which other barriers to access are addressed. Only if additional efforts are directed towards addressing non-financial barriers to access, will high coverage levels be achieved and sustained.

## Background

Ensuring that the poor and vulnerable population benefit from malaria control interventions remains a challenge for malaria endemic countries. Insecticides Treated Nets (ITNs) are the most effective means of preventing malaria [1-5]; they provide significant protection against early childhood mortality under a range of malaria settings in Africa, and reduce the incidence of clinical malaria and anaemia in young children [6,7]. The cost-effectiveness of ITNs relative to other malaria control interventions has also been demonstrated [8-11]. A major challenge for malaria control in the last decade has been how to improve ITNs coverage among young children and pregnant women. In the Abuja declaration of 2000, African heads of states committed to ensure that ITNs are available to 60% of children under five years and pregnant women by 2005, a target that has since been revised to 80% by 2010 [12].

Until recently, most African countries recorded low levels of ITN coverage and wide inequities between the poor and the rich existed [13-16]. Between 1998 and 2002, ITN use among children under five years was less than 5% in 23 countries [17]. Low coverage levels were attributed to existing delivery strategies which mainly comprised of the commercial retail sector and social marketing. Alternative distribution strategies were urgently required to facilitate progress towards the Abuja targets. A popular quick win strategy was the integration of ITN distribution into mass vaccination campaigns [12]. Mass vaccination campaigns typically reach 90% of their target population [18] hence they provide a unique opportunity for rapid increase in ITN coverage. Questions on the sustainability of mass distribution strategies were hardly explored [19], or at least they were not documented.

Reassuringly, ITNs coverage has improved dramatically in many settings across Africa where free distribution was integrated into mass vaccination campaigns. In Kenya, ITN ownership in four malaria endemic districts increased from 23.5% in 2005, to 67.3% in 2006 following two mass distribution campaigns [2]. Wealth related inequalities that previously existed were eliminated. Coverage levels among children in the poorest quintile increased from 17.5% in 2005/2006 to 66.3% in 2006/2007, the highest increase recorded [2]. A nationwide evaluation however reported that only 39.2% of children aged below five years slept under an ITN on the night preceding the survey [20], indicating that the mass campaigns were not as successful in other parts of the country. In Zambia, ITN ownership increased from 16.7% prior to a mass campaign to 81.1%, although the rate of use was below 60% in the rural areas [21]. High and equitable coverage levels were also reported in Mozambique, Ghana, Niger and Togo [21-23].

Although the high coverage indicates a major success for malaria control, usage levels remain below the Abuja targets [2,20,23,24]. Reducing financial barriers is necessary, but not sufficient, as there are other equally important determinants of access, such as community preferences for different ITN designs, perceptions of ITNs, the types of ITNs available in the market, household size and structure [25-30]. These factors are not addressed through free provision of ITNs. Moreover, mass campaigns improve socioeconomic and geographic equity but are inefficient in achieving timing equity, i.e. children born shortly before a campaign enjoy far better health benefits than children born later in the inter campaign interval [31].

### Delivery of ITNs in Kenya

Noor *et al *provide an overview of ITN delivery strategies in Kenya and how the scenario has changed over time. ITN distribution strategies over the years have included [2]: (1) partially subsidized ITNs distributed through a social marketing approach by the Population Services International (PSI) with support from the UK Department for International Development (DFID). This distribution approach was the main source of ITNs in 2002-2004 and targeted urban and rural retailers in malaria endemic districts; (2) heavily subsidized ITNs sold to pregnant women and children under five through maternal and child health clinics. This approach was initiated in 2004 through a partnership between the Ministry of Health and PSI, and received financial support from DFID; (3) free distribution of ITNs to pregnant women and children through the public health sector and; (4) the commercial retail sector, where retailers sell ITNs for profit. Other short term distribution strategies include project specific or distribution by non-governmental organizations (NGOs) and the mass distribution campaigns conducted in 2006. The mass campaigns were conducted in two phases: the first phase was integrated with a mass measles vaccination campaign and the second was a stand-alone campaign conducted by the Division of Malaria Control (DOMC). The campaigns were funded through a US$ 17 million awarded by the Global Fund to Fight AIDS, TB and Malaria (GFATM) to support the procurement and distribution of 3.4 million Long Lasting Insecticides Nets (LLINs) free of charge to children aged below five years.

At the time the study described in this paper was initiated in 2005, plans began for the 2006 mass distribution campaigns. Dramatic increases in coverage were expected; but we hypothesized that the poorest of the poor would not benefit as much from the interventions as the wealthiest groups. Although a dramatic increase in ITN coverage was recorded for all socio-economic groups in four districts, a third of children under five were not reached through the interventions [2], and national level coverage remain far below the Abuja targets [20]. In addition, mass campaigns are periodic and are often linked to vaccination campaigns that target specific age groups, and therefore potentially miss important groups including pregnant women, very young children and children born after the campaign. Understanding barriers to access among the poorest of the poor therefore remains important.

The study was conducted in the poorest malaria endemic areas in Kenya [32] to explore barriers to ownership and use of ITNs among the poorest populations before and after the campaigns, to identify strategies for improving coverage and to make recommendations on how to sustain high coverage levels. The paper focuses on the general distribution of ITNs but also draws on experiences from the mass campaigns. It adopts a new analytical framework [33] and explores demand, supply and policy level barriers to access. It also demonstrates how factors related to the different dimensions interact to influence ownership and use of ITNs. A comprehensive understanding of these factors is required for generating concrete and actionable policy recommendations.

### A framework for exploring barriers of access to ownership and use of ITNs

**Figure 1:**
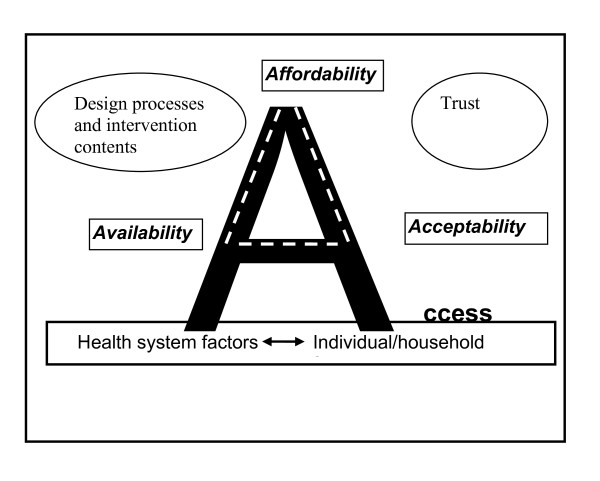
**Analytical framework**. Source: adopted from Thiede et al., 2007.

Various definitions of access exist [34-37]. Common across these definitions is the recognition that access is a multidimensional concept, affected by interrelated factors, occurring at both the demand and supply side. Recently a comprehensive framework that highlights three distinct yet interrelated dimensions of access has been developed [33,36]. According to the framework, access can be measured using three dimensions (Figure 1): (1) Affordability (sometimes referred to as financial access) and includes the costs of seeking care and ability to cope; (2) Availability (also known as physical access), referring to the geographical location of health care services in relation to the clients and; (3) Acceptability, referring to the nature of service provision and how individuals and communities perceive it. Acceptability includes the social and cultural aspects of access.

Central to the framework is the role of information in promoting access. Information cuts across the three dimensions and is considered a prerequisite for promoting good interactions between the health system, individuals and communities. It empowers individuals to make well-informed decisions about health services use. Although the framework was developed and has been applied to measure access to health care services, the study adopts the framework to explore access barriers to ITNs. Two additional factors important for understanding barriers to ITNs are highlighted: (1) the design process and contents of an intervention and; (2) trust in agencies involved in ITN delivery.

## Methods

### Study setting

The study was conducted in four districts in Kenya, purposively selected to represent the country's different ecologies and malaria transmission patterns. Kwale district on the coast with seasonal, high intensity transmission; Bondo on the shores of Lake Victoria with high intensity perennial malaria transmission, Gucha district representing the low seasonal transmission conditions of the Western highlands; and Makueni, a semi arid district with acutely seasonal low transmission. Also considered in the study site selection was the existence of a wide range of research activities conducted by colleagues in KEMRI-Wellcome Trust Research Programme in collaboration with the DOMC to monitor changes in ITN ownership and progress towards Abuja targets [1-5].

The districts had different experiences with free or subsidized ITNs distribution prior to the mass campaigns. A research organization had distributed free ITNs to most households in Bondo district, while an NGO working in Gucha district occasionally provided free ITNs to vulnerable groups including children under five and orphans. Kwale and Makueni districts had no history of a large scale free ITN distribution prior to the mass campaigns. ITN coverage levels were low in all districts prior to the campaigns. In 2004 the proportion of children aged below five years who slept under an ITN was less than 20 per cent in each of the districts [38].

The districts are also some of the poorest in the country [32]. The percentage of individuals living below the poverty line is estimated as 63% in Kwale, 62% in Makueni, 61% in Gucha and, 70% in Bondo [32]. As discussed below, we were interested in the lowest income locations within these districts because of past and expected wealth related inequalities in access to ITNs.

### Data collection methods

Data were collected in two phases before and after the campaign to enable comparison in ITN ownership. A cross-section survey with 708 households was conducted in April to June 2006 (pre-campaigns) to gather data on socio-economic characteristics, ITN coverage and to provide quantitative information on factors influencing ownership of ITNs. In-depth interviews with key informants were also conducted prior to the campaign. A second survey was conducted after the mass campaigns (January to March 2007) to record changes in ITNs ownership and use. A semi-structured questionnaire was administered in the local language to the household heads or spouses or, in their absence, another senior member of the household. All respondents were aged above eighteen years.

A multi-stage sampling approach was adopted to select the survey households:

• First, locations (the 2^nd ^lowest administrative unit in Kenya) were selected using poverty indicator maps. The maps classify locations within each district into poverty quintiles based on the percentage of the population living below the international poverty line of USD 1 per day [32]. All locations that fell within the two poorest quintiles in each district were identified. Focusing on the two poorest quintiles increased the chances of reaching the poorest households.

• Four enumeration areas (EAs) in the two poorest quintiles in each district were randomly selected. The EAs had been mapped in 2001 as part of the various research activities conducted in the districts by our colleagues in collaboration with the DOMC.

• Homestead lists were updated and a total of 100 homesteads were randomly selected from the participating EAs. A homestead was defined as a collection of adjacent or nearby households with a single individual as an administrative head.

• All households in the homestead were included in the study resulting to a total of 708 households. A household was defined as a group of people living in the same area and who share a common source of food and/or income. There could have been more than one household within a homestead, because residents did not always share income, or eat from the same pot.

FGDs and semi-structured interviews with ITN suppliers were conducted after the mass campaigns. These data collection methods provided an opportunity to explore access barriers in more detail, enabling the researchers to gather additional information that would not have been captured in the quantitative survey. The FGDs also provided an opportunity to explore how barriers related to the three dimensions of access interact to influence ITN ownership at the household level. Key topics covered in the FGDs included perceptions on malaria causes, sources of ITNs, net use patterns, barriers to ITN ownership and use and how they can be addressed, experiences with the mass campaigns distributions and economic generating activities. Data were collected up to a point of redundancy (n = 24 FGDs). FGD participants were selected on the basis of age, gender, and place of residence to allow for diversity. Priority was given to men and women in their reproductive age because children are more vulnerable to malaria in the study settings [39]. Older men and women were also considered due to their important role in treatment seeking decisions [40]. All FGDs were tape recorded and notes taken to supplement the recorded information.

To capture supplier related barriers to access, additional data were collected from two of the four districts (Kwale and Makueni). The two districts were chosen because they were the poorest of the four [32], and poverty was an important theme in the study. Semi-structured interviews were conducted with different types of ITN suppliers including: 27 commercial retail outlets; 32 public primary health care facilities (dispensaries and health centres) and 11 NGOs. The suppliers were selected purposively and through snowballing. Key topics covered in the interviews included types of ITNs normally in stock, sources and challenges around delivery mechanisms; perceptions on affordability and acceptability of ITNs in the community they served, the role of the mass campaigns in improving coverage and perceptions around the strengths and weaknesses of the campaigns.

### Data analysis

Quantitative data were double entered into Microsoft visual FoxPro (version 9.0) and transferred to STATA (version 9.2) for analysis. Data were analyzed for both ITN ownership and use. Ownership was assed at a household level, while ITN usage focused on children aged below five years. Total monthly expenditure was estimated by summing up monthly spending data on 14 items namely: food, cooking fuel, cleaning, lighting, rent, transport, remittance, education, debt repayment, contribution to community groups and churches. Expenditure data were converted into per capita estimates by dividing total monthly expenditure by household size.

FGDs data were transcribed and transferred to Nvivo 7.0 for analysis. Data were coded and themes and sub-themes identified by two investigators to ensure trustworthiness. Data from different sources were analysed to enable triangulation.

### Ethical issues

Informed consent was sought from all study participants. Ethical approval was obtained from the Kenya Medical Research Institute (SCC No. 964), and from the World Health Organisation Research Ethics Review Committee (ID A50045).

## Results

### Description of study participants and ITN suppliers

Survey respondents were mainly household heads (58.1%), or their spouses (35.9%). Most respondents had attended school (72.6%), although only 20.0% had progressed beyond primary school. Drawing on the pre-campaign survey, results presented in Table 1 show that education levels were relatively low in the districts; 24% of adults had never attended school and; only 25% had beyond primary school education. Agriculture was the main source of income for household heads (70.3%). Mean monthly per capita expenditure was below the internationally accepted poverty line of US$1.25 per day (US$ 15.4 per month). The main sources of ITNs were government health facilities (65.9%) and the retail sector (16.9%). Lack of money was the main factor that prevented households from owning ITNs (88.5%). Other factors given included ITNs were not readily available in the market (8.6%) and that ITNs were not necessary (5.8%).

**Table 1:** Characteristics of survey households

Variable	n = 708
Mean (range) household size	6 (2-37)
Number of children under five	729 (17.3)
Adults with education > primary school	447 (24.8)

Main occupation of household head	
Agriculture (small scale)	498 (70.3)
Casual labourer	76 (10.8)
Petty trade	64 (9.0)
Other	70 (9.9)

Median monthly per capita expenditure in KES (USD)	1202 (15.4)

How long ago acquired last net	
< 1 year	240 (52.1)
1 year	84 (18.2)
2 years	44 (9.5)
3 +years	93 (21.0)

Net conditions from households perspective	
Good condition	219 (47.4)
Poor condition-torn but usable	115 (24.9)
Very poor condition-not usable	9 (2.0)
Differs for different nets	119 (25.7)

Potential source of ITNs	
Local shop/market	120 (16.9)
Government dispensary	404 (57.1)
Government hospital	62 (8.8)
Community Health worker	22 (3.1)
Faith based health facility	22 (3.1)
Do not know	36 (5.1)
Other	42 (6.0)

Main reasons for not owning ITNs (%)	
Not necessary	14 (5.8)
Expensive/cannot afford	216 (88.5)
Nets not available	21 (8.6)
Other	13 (5.3)

Table 2 shows the number of households that owned ITNs before and after the mass campaigns. The results indicate that about two thirds of the households owned at least one ITN prior to the campaigns, but only 50.1% of children aged below five years slept under an ITN on the night before survey (Table 3). The proportion of households owning at least one ITN in the pre-campaign survey was highest in Bondo (89.3%) and lowest in Kwale (47.8%). There was a dramatic increase in the proportion of households owning ITNs in all districts after the mass campaigns (p < 0.001), and in the proportion of children under five that slept under ITNs (p < 0.001). However, the increase in the proportion of children who slept under an ITN on the night before the post-campaign survey was not as high in Gucha (p = 0.053) and Kwale (p = 0.006) districts.

**Table 2:** Net ownership among survey households

District	Number (%)of households that owned < = 1 net	95% Confidence Interval	P-value
Bondo			
Pre-campaigns (n = 179)	159 (89.3)	84.2 - 93.4	0.038
Post-campaigns (n = 146)	139 (95.2)	91.7 - 98.7	

Gucha			
Pre-campaigns (n = 204)	140 (68.7)	62.3 - 75.0	0.001
Post-campaigns (n = 175)	145 (82.8)	77.3 - 88.4	

Makueni			
Pre-campaigns (n = 141)	76 (53.9)	45.7 - 62.1	0.00
Post-campaigns (n = 130)	106 (81.6)	74.9 - 88.2	

Kwale			
Pre-campaigns (n = 184)	88 (47.8)	40.6 - 55.0	0.00
Post-campaigns (n = 174)	129 (74.3)	67.6 - 80.6	

All districts			
Pre-campaigns (n = 708)	463 (65.4)	61.9 - 68.9	0.00
Post-campaigns (n = 625)	519 (83.1)	80.0 - 86.0	

**Table 3:** Proportion of children aged 5 years that slept under a net on the night preceding the surveys

District	Number (%)of children under five that slept under ITN night preceding survey	95% Confidence Interval	P-value
Bondo			
Pre-campaigns (n = 141)	95 (67.4)	60.0 - 75.0	
Post-campaigns (n = 135)	108 (80.0)	73.3 - 86.7	0.02

Gucha			
Pre-campaigns (n = 198)	114 (57.6)	50.7 - 64.5	0.05
Post-campaigns (n = 180)	121 (67.2)	60.4 - 74.1	

Makueni			
Pre-campaigns (n = 138)	64 (46.4)	38.1 - 54.7	0.00
Post-campaigns (n = 129)	95 (73.6)	66.0 - 81.2	

Kwale			
Pre-campaigns (n = 252)	92 (36.5)	30.6 - 42.5	
Post-campaigns (n = 266)	129 (48.5)	42.5 - 54.5	0.006

All districts			
Pre-campaigns (n = 729)	365 (50.1)	46.4 - 53.7	0.00
Post-campaigns (n = 710)	453 (63.8)	60.3 - 67.3	

Public health care facilities, NGOs and the commercial sector were the main sources of ITNs in the districts (Table 4). The ITNs supplied differed in shape and colour. Dispensaries and health centres usually stocked rectangle ITNs, which were either blue or white depending on the source, while NGOs and Community Based Organisations (CBOs) distributed either green or blue ITNs and included both round and rectangle nets. Public health care facilities (dispensaries and health centres), and some NGOs distributed ITNs free of charge when they had some available. Otherwise, the price of ITNs ranged from KES 20 (for CBOs) to KES 450 (for the commercial retail sector). The main source of ITNs for dispensaries and health centres were the district hospital and PSI. NGOs sourced ITNs from their headquarters, while CBOs either acquired ITNs from NGOs working in the settings or sourced them from PSI. Public health facilities also engaged CBOs in ITNs distribution.

**Table 4:** ITNs suppliers and the types of nets available in the study settings

Supplier	Sources of ITNs	Shape	Colour	Price
Public health facilities (Dispensaries and health centres)	District hospital PSI	Rectangle	White	Free when available
		Rectangle	Blue	KES 50

NGOs	NGOs head quarters	Rectangle and Round	Blue, White and Green	Free

CBOs	NGOs, health facilities, manufacturers	Rectangle and Round	Green and blue	KES 20 to 200 depending on source

Commercial retail sector	Manufacturers, wholesalers and NGOs (PSI)	Round and Rectangle	Blue and White	KES 300 to 450

### Barriers to ownership and use of ITNs

#### Affordability related factors

On the demand side, affordability is well understood to be an important factor affecting access to ITNs; recognition which has contributed to mass distribution campaigns in Kenya and elsewhere. The survey data on reasons for not owning a bed net support that affordability was key before the campaigns. Affordability was raised in all FGDs as a barrier to access. Affordability barriers were attributed primarily to the actual cost of purchasing ITNs from different sources, but given the relatively remote location of some of these households, the indirect costs (transport, work lost in going to a central place to buy or wait for a bed net), appeared also to play a role:

"Only those with money, like those who have a salary every month can get an extra KES 100 to buy a net. For us who work on the farms, the money we get is only enough to buy food for the children" (FGD, Women)

"The main thing is lack of money. People who do not have nets now once had them, but they have grown old and there is no money to buy others" (FGD, Men)

While affordability was reported as a barrier to access in all FGDs, even after the campaigns, interviewees were also quick to point out that other factors related to acceptability and availability are also important determinants of access; in some cases more important barriers:

"We do not agree. It is not only the money...if it is the money why do people refuse to go for free nets?" (FGD, Men)

On the supply side, affordability factors included the cost of buying ITNs from wholesalers or manufacturers. The cost of acquiring ITNs was reported to be relatively high, and while the willingness to continue selling ITNs existed, the high cost of purchasing ITNs limited the suppliers' ability to stock them. Public health facilities in particular expressed concerns regarding shortage of funds to sustain ITN programmes following a policy change that reduced user fees significantly, and low sustainability of donor supported programmes:

"Getting money to buy the nets is a problem because we depend on the little amount collected through the user fees." (Health worker)

"They [referring to a donor] helped us to start the ITN project. They sold the nets to us at KES 15, although the actual price was KES 30. We then sold the nets to the community at KES 50 and made some profit. But this support came to an end without any explanation. We do not know what happened and we cannot get these nets again." (Health worker)

#### Acceptability related factors

Community preferences and beliefs were identified primarily through qualitative work as important demand side determinants of access. Although people had a range of different preferences for ITNs, in general 'non-white' nets were reportedly preferred in all districts. White nets easily got dirty and were regularly associated with misfortunes, including bad dreams and deaths. Round ITNs were reported as easy to hang although they were uncomfortable to use during the hot season, while rectangular nets were cumbersome to hang but they enabled air circulation if they were large enough. Participants in 17 FGDs expressed concerns that community preferences were not considered in the design of interventions. In particular, it was reported that the rectangular ITNs distributed through the mass campaigns resembled a coffin, and that their white colour resembled a burial shrewd. It was reported in about half of the FGDs that some households who received free ITNs through the mass campaigns apparently did not use them. They were either returned to the distribution centres, thrown away, or used for other activities like fishing:

"If you are an adult who has never slept under a net all your life, then out of nowhere someone brings you a free white net, you have every reason to belief that you will die. A white net symbolises death. Sleeping under it invites death in the family" (FGD, Men)

"Many of us returned those nets [referring to ITNs distributed through the mass campaigns] to the dispensary at night, others burnt them and others tore them into pieces and used them to catch fish...that is the truth." (FGD, Men)

Community members reported that while they did not always use the ITNs provided through campaigns or through public health services for the intended purposes, this information can be hidden from researchers or outsiders:

"There is this time the government brought free nets and most of us got them [referring to ITNs distributed during mass campaigns]. However, the worst thing is that most of the people who got them in this village do not use them. Instead, they tear them up and use them as curtains or blankets. But when you people come to our homes to ask questions, we will not tell you that we have turned the nets into curtains or that we do not use them." (FGD, Men)

Health workers also expressed their concerns regarding community perceptions and their impact on uptake and acceptability of ITNs:

"Acceptability is not so good these days...the nets given for free are even fishing in the ocean...the rumours have really affected net usage here and how people perceive nets...especially white rectangular nets." (Health worker)

Another concern related to acceptability was targeting of women and young children. Community members were generally aware that pregnant women and children below five years are the most vulnerable to malaria. However, the link between targeting interventions and vulnerability was not always made or clear. Mistrust and suspicions regarding the reasons for targeting were reported in 11 FGDs, with people expressing fears that free ITNs would destroy the future generation. To the community, a useful commodity should be given to all irrespective of age or gender. The importance of information in minimising rumours and suspicions was also highlighted:

"Some people are suspicious that nets could kill people because they are free and are given to specific groups. If nets are good as they say, why are they not given to everybody?" (FGD, Women)

"We should be told or educated why it is only the women and children under five who are given free nets... that way people will stop being suspicious." (FGD, Men)

Other acceptability factors often grouped in the literature under social cultural barriers were illness perceptions and treatment seeking behaviour. Household size and structure were other important determinants of ITN ownership and use. It was reported that it was not always possible to acquire enough ITNs to cover all children within a household, and that children often slept on mats spread either in the common room or in the kitchen. Covering children sleeping on mats with ITNs was reported to be cumbersome and sometimes impossible, particularly when the nets were too short:

"It is the adults who use the nets instead of the children because children sleep in groups on a mat. It is also difficult to hang a net over a large mat. If you hang it on the roof, the net is too short, if you fix it on the wall it covers only half of the mat...and there is no large enough net to cover a large mat with a group of children." (FGD, Women)

"In most homes, people use tin lamps and because nets catch fire easily, people fear that it can be disastrous to have a net in the house...especially when the net is small and with children around." (FGD, Men)

Gender featured quite strongly in discussions, with women feeling that health education was targeted towards women, yet men control resources and are often the main decision makers in the households. Women therefore highlighted the need to involve men in malaria control:

"Another thing, our husbands always ignore these issues because when you tell him about buying a net for the child, he tells you to find your own means of buying a net to protect that child of yours...you see he leaves the child to you, that it is yours not his. If we [women] do not have the money, the nets get finished and we miss out. The other thing is that, you people talk to the women. How will the men know that nets are important, yet they are the ones who have the money? If they are enlightened through health seminars, they will know that nets are important, and then they can buy the nets for us and for the children." (FGD, Women)

"My husband says, 'I can see you are surrounding me with a net, if people start shouting out there for help, how easy is it going to be for me to come out of it?' But you know the child [who shares a bed with parents] always suffers from malaria and the father does not want to sleep under a net. Now tell me, how can I solve this? And when the child gets malaria and is crying, the father covers himself with a blanket and sleeps deeply." (FGD, Women)

#### Availability related factors

The types of ITNs available in the market and the location of centres that sell them in relation to the location of the community influences access. Participants in 10 FGDs reported that ITN retailers are generally located in towns, while public health facilities are sparsely distributed and accessible only by poor roads which are impassable during the wet season:

"We did not get the free nets [referring to the mass campaigns] because the roads are very bad and the nets were issued in the rainy season. You have been here many times [referring to the research team] and you have seen the state of our roads. From the main road to the village, there is no road and where you passed today was a bush, we just cleared it the other day to make something that looks like a road. We can do that as a community, but we cannot build a bridge across the river." (FGD, Men)

Health workers expressed their willingness to deliver ITNs to the community, but lack of transport and poor infrastructure made them reluctant to distribute ITNs in the remotest areas of the districts. Some health workers could not distribute ITNs provided through the mass campaigns to the remotest areas due to lack of transport:

"The distance to the outreach centre is long, the roads are rough and we travel by a motorcycle. How many nets can one carry on a motorcycle?" (Health worker)

Even when ITNs were available, the limited variety impacted negatively on access. Subsidised ITNs provided through primary health care facilities or through other interventions were usually standard in shape, colour and size and did not always match people's demands:

*"The nets available at the dispensary and the ones issued for free are square...they have four corners but they are not big enough...they are too short...they cannot reach the ground to cover children sleeping on a mat. Another thing is that some of us live in small houses where we also do the cooking. It makes us feel that since the net is white, the smoke will make it dirty...so we do not use it" (FGD, Women*)

Interviews with ITNs suppliers yielded similar results with many expressing concerns about the impact of limited choice on acceptability of free and subsidised ITNs:

"Many community members prefer round nets which are not available. When they come here, they will first ask if we have round nets...But that blue, square, net is what everybody gets. Others say they cannot pay KES 50 for a net that is too small [referring to the blue square net available in government facilities for KES 50 and sometimes for free" (Health Worker)

On the supply side, the location of ITNs manufacturers and wholesalers was identified as a barrier to access. Most ITNs manufacturers, wholesalers and distributors are located in urban areas, and while some deliver ITNs to rural areas, they are often reported as unreliable. Subsidised ITNs distributed through the public health sector were often delivered to district hospitals and primary health care facilities made their own arrangements to collect them from the district. Often this required transport and such funds were not always available:

"We usually contact the person in charge of supplying bed nets at PSI when the nets are over but he does not come immediately, it may take even months. We may go to the district hospital but the district hospital does not provide transport and most of the times we do not have money to transport the nets ourselves." (Health worker)

Market competition was reported as a challenge for the retail sector. Subsidised ITNs available in the public sector (ideally meant for the vulnerable groups), were sold to anyone who could afford them, rendering the services of retailers unattractive. Community members acknowledged that they were unlikely to buy ITNs from the retail sector when they knew they could 'illegally' acquire subsidised ITNs from the public health sector. Consequently, most retailers had stopped selling ITNs, while those that had ITNs in stock were unwilling to re-stock their shops because ITNs were taking too long to sell:

"Our businesses have been affected by the cheap and free nets being given out at the health facilities. No one buys nets from us anymore...so it becomes difficult for us to stock nets when no one is buying them. People know they can get cheaper ones from the dispensary." (Retailer)

Some people can afford to buy nets from the shops, but here is a situation where I can bribe those who give free nets with KES 20 to give me five nets. Why then should I bother to buy one from the shop? Therefore, we are the ones who kill government programmes through our corruption and we also kill the shops (FGD, Men)

The availability of subsidised ITNs to the larger population presented a leakage in the distribution system. Health workers and other individuals entrusted with the distribution of subsidised or free ITNs did not always adhere to the distribution guidelines. In almost all FGDs, participants reported that free ITNs were sold, and prices for subsidised ones increased. Selling free ITNs or increasing prices for the heavily subsidised ones posed an affordability barrier and undermined the potential for reaching the poorest groups with interventions:

"I speak as a dispensary committee member. The health worker stopped issuing free nets and instructed the watchman to sell the remaining nets through the rear window. Most mothers who had come to collect the free nets and did not have the money required were not given a net." (FGD, Men)

"Recently free nets were distributed but the people in charge demanded KES 10 from each potential beneficiary. They said it was for their lunch...those who did not have money did not get nets, while those who ran back home to get the money returned when nets had run out." (FGD, Women)

Interviews with health workers confirmed these concerns, but they also revealed the reasons why they did not always adhere to guidelines. Although health workers understood the importance of targeting, they did not always provide ITNs to vulnerable groups due to various reasons including: limited storage capacity; low sales resulting to lower profits especially where facilities used their own funds to stock ITNs; pressure from other community members to sell ITNs to everyone and; to help minimise suspicion and rumours and in the process address demand side barriers related to acceptability:

"*Although the KES 50 nets are provided for children under five and pregnant women, we sell them to everybody because the dispensary health committee wants to make money, and again the community complains so much that they also need the nets. When the nets were reserved for children less than five years the community was suspicious of the motive behind the targeting...we had to sell the nets to others in the community to prove that there was no ill motive in targeting the young children." (Health worker)*

Other supply side challenges often included high workload that burdens already overstretched health workers. Health workers reported that it was difficult to fully provide ITNs under existing programme rules and regulations. They failed to distribute ITNs during their routine health outreaches in remote areas because they found it cumbersome to keep records for their outreaches-as part of their job- and for ITN sales which was often seen as an extra activity.

## Discussion

This study set out to explore access barriers to ITNs ownership and use among the poorest groups in Kenya. High coverage levels were achieved with the mass ITN distribution campaigns in the study districts, including substantial increases among low income groups [2]. A national level survey reported much lower coverage of 39.2 percent among children under five [20]. Sharp improvements were noted in our study, although in Gucha and Kwale districts, these were not as dramatic as reported by Noor *et al*. There are many potential explanations for these differences in coverage levels, including the deliberate selection of the lowest income locations within districts in our study, our relatively small sample size, methodology differences in estimating socio-economic status, and differences in definition of households. Regardless, our findings support those by Noor *et al *[2], and highlight that understanding barriers to access among the poorest of the poor remains important, irrespective of the coverage levels.

**Figure 2:**
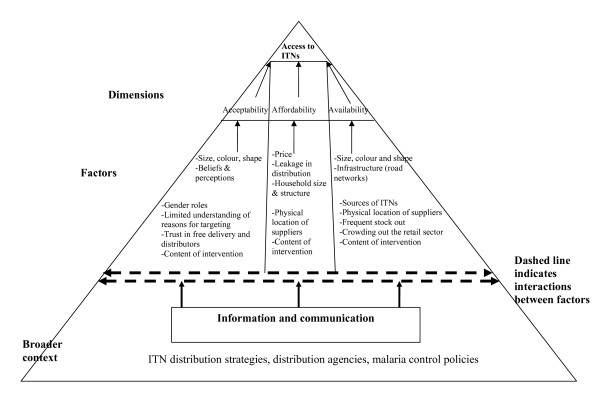
**A summary of factors influencing access to ITNs ownership and use**. Source: adopted from McIntyre et al., 2009.

Through using the access framework, we identified a range of demand and supply level factors that cross-cut across the three dimensions of access to influence ownership and use of ITNs. Figure 2 presents a summary of the cross-cutting factors influencing access to ITNs.

Although the surveys identified affordability as the main barrier to access, qualitative data suggested that other barriers related to acceptability and availability were significant determinants of access, including on affordability itself. Size, colour and shape of ITNs were major access barriers. Perceptions on ITNs differed by these characteristics, with most people preferring non-white and round nets, which were hardly provided through interventions. Previous studies conducted in different parts of Africa yielded similar findings [27-29], although availability of ITNs has hardly been documented as a barrier to access. In two of the districts, there was a widely held belief that free ITNs distributed during the mass campaign brought misfortunes and deaths. Not surprising, these districts recorded the lowest levels of ITN use. These findings suggest that perceptions and beliefs are important determinants of access; but they also demonstrate a general lack of trust in free delivery and in the government and agencies involved in ITN distribution.

Negative perceptions on ITNs distributed as part of an intervention can impact on affordability and availability. While governments and donors may be willing to subsidise ITNs, low acceptability of subsidised ITNs limits the community's potential to benefit from interventions, implying that those who need ITNs continue to buy them at a higher price from more trustworthy and acceptable sources. This would often require additional costs that may have been unnecessary if the subsidised ITNs matched communities' needs, and if the community trusted the distributing agency. Incorporating community preferences when designing interventions (for example through ensuring that people can choose between round and rectangular nets, white and non-white nets, short or long nets), providing timely and adequate information can help address some of the concerns identified in this study, minimise rumours and suspicions, strengthen trust in the distribution agencies and ultimately promote acceptability and ITNs use.

Gender differences in access to resources can hinder ITN ownership and use. In our study settings, as elsewhere in sub-Saharan Africa, men control resources and are the main decision makers in the households [41]. Concerns were expressed that information regarding malaria control interventions is often given to women mainly during maternal and child health clinics, yet it is the men who have money and make decisions on whether or not to buy ITNs. Engaging men more actively in malaria control through education can empower them to buy ITNs when resources are available, and be more willing to use them when they share a bed with a young child.

On the supply side, challenges differed by type of supplier. For public health care facilities, concerns were around the infrequent supply of subsidized ITNs from the government and donors, and the fact that the type of ITNs provided were not liked by the community they served. Sustained supply of subsidized ITNs to the public health sector; supporting dispensaries and health centre to stock subsidized ITNs; delivering ITNs to the local facilities in addition to the district; and providing different types of ITNs can help improve availability and strengthen acceptability.

For the retail sector, the major concern was that free and subsidized ITNs were crowding retailers out of the market due to the leakage in the distribution system. Crowding out of the retail sector has various implications for malaria control. For example, in the absence of the commercial retail sector, ITNs may not always be available to those who can afford to buy them at a higher price, and people may have to travel long distances to purchase ITNs, often incurring high transport costs. While these findings might be unique to remote rural settings, and the urban commercial sector might not be threatened by the existence of alternative distribution strategies, they highlight an important issue that has not been previously documented in the literature, but which requires urgent attention. It is evident that ITNs sold through the retail sector do not reach the poor [2], and that free distribution eliminates inequities [2,21,24,42]. However, mass campaigns work better when combined with regular 'keep-up' strategies to provide ITNs to those who miss out on campaigns and to children born after the campaigns [42]. The retail sector can be a useful 'keep-up' strategy for those who can afford to pay, those who miss out on interventions, and those who do not use the public health care system. Protecting the retail sector is complex and subject to debate due to its profit maximizing goal, but a combination of distribution strategies are required for sustaining and improving the high coverage levels already achieved [2,42]. Potential ways to support the retail sector in remote rural areas might include providing subsidised ITNs to retailers in malaria endemic areas and that the government and donors support the delivery of these ITNs to minimise the costs incurred by retailers. Such delivery approaches previously existed in Kenya, and while they might not have been effective in reaching the poor, they provided a sustainable market for those who could afford to pay. The Kenyan government and donors should consider reintroducing subsidised ITNs to retailers as a 'keep-up' strategy. Only if a combination of distribution strategies is maintained will high coverage levels be achieved and sustained.

The role of infrastructure in promoting or hindering access to ITNs should be emphasised. Interviews with ITN suppliers and community members indicated that poor roads were major barriers to access. Improving infrastructure may ensure that manufacturers or wholesalers are more willing to deliver ITNs to retailers and health care facilities in remote settings; that health workers can supply ITNs during their routine outreach programmes; and that community members can travel with ease to outreach and market centres where ITNs are readily available. Access to markets has been shown to influence ITN ownership and use [30,43,44]. Others have noted that improving road quality can have a significant impact on ITNs ownership as health education campaigns or financial subsidies, and that more efforts should go into infrastructure development [30]. Investing in road networks is beyond the scope of Ministries of Health. Nevertheless, improving access to malaria control and health care in general requires multi-sectoral action.

An important factor cutting across all access dimensions is information. Information empowers communities to use health care services [36,45], it shapes peoples thoughts and actions. Lack of information and poor communication regarding ITN related interventions in the study community hindered people from owning and using ITNs. Specifically, the community did not understand the link between vulnerability and targeting and no attempts were made to inform them as to why the mass distribution campaigns and other interventions targeted children under five and pregnant women who culturally represent the future generation. This lack of information, together with poor communication between the community, the people responsible for designing interventions, and the implementers was partly responsible for the rumours and suspicions associated with the mass campaigns. Poor communication practices and inadequate preparations of the community prior to an intervention may impact negatively on perceptions and on intended gains from the intervention. Addressing the barriers to access identified in this study will require significant efforts to improve communication and information flow. Such messages should be informed by research and tailored towards community needs.

### Limitations

It is possible that some of the bed nets under use were not ITNs. One would expect barriers to access to be similar for both treated and untreated bed nets and thus the findings remain relevant irrespective of this limitation. Second, the findings are based on perceptions which cannot be easily validated. Nevertheless, perceptions influence behaviour and impact on access. Thirdly, the study did not measure the magnitude of the access barriers quantitatively. While this was not the aim, future studies should attempt to quantify some of the barriers identified in this study. Fourthly, these findings are not generalisable to the wider population. The aim was to understand barriers to access among the poorest groups in detail, and while the findings might be unique to the study contexts, lessons drawn from this study can inform the design of interventions elsewhere. Fifthly, people's perceptions can be influenced by many factors and it is possible that some people might have reported negative experiences with ITN distribution because they did not benefit from subsidised or free ITNs. Finally, data were collected in the poorest settings of four districts. Barriers to access may differ in more developed settings, in urban areas with better infrastructure, and where the commercial ITN sector is more developed. Nonetheless, important lessons on how to improve access to ITNs can be drawn from the findings.

## Conclusions

It is clear that multiple barriers to ITN access exist. Significant resources have been directed towards addressing affordability barriers through free provision of ITNs to vulnerable groups. Providing free or heavily subsidised ITNs is essential, but the success of these interventions depends largely on the degree to which other barriers identified in this paper are addressed. The challenge remains how to ensure that barriers related to the different access dimensions are incorporated in the design of interventions. Finally, implementing the recommendations highlighted in this paper requires additional resources (financial and time) be directed towards malaria control. Governments, donors, bilateral and international organisations, are largely responsible for ensuring that the goal of reaching 80 percent of the vulnerable population by 2010 is achieved, and that increased coverage levels are sustained.

## Competing interests

The authors declare that they have no competing interests.

## Authors' contributions

JC and CM were involved in the conception and design of the study. JC, JN, VO participated in data collection, analysis and writing up. JC wrote the first draft; all authors commented on drafts, read and approved the final manuscript.

## Pre-publication history

The pre-publication history for this paper can be accessed here:

http://www.biomedcentral.com/1471-2458/10/137/prepub
